# Supplementary material for: Genetic loci for lung function in Japanese adults with adjustment for exhaled nitric oxide levels as airway inflammation indicator
Source: Commun Biol. 2021 Nov 15;4:1288. doi: 10.1038/s42003-021-02813-8 (PMC8593164; doi:10.1038/s42003-021-02813-8)
Supplement: Supplementary file 2 — Supplementary Information [file 42003_2021_2813_MOESM2_ESM.pdf]

Supplementary Information

Genetic loci for lung function in Japanese adults with adjustment for exhaled nitric oxide levels as airway inflammation indicator

Mitsuhiro Yamada, Ikuko N. Motoike, Kaname Kojima, Nobuo Fuse, Atsushi Hozawa, Shinichi Kuriyama, Fumiki Katsuoka, Shu Tadaka, Matsuyuki Shirota, Miyuki Sakurai, Tomohiro Nakamura, Yohei Hamanaka, Kichiya Suzuki, Junichi Sugawara, Soichi Ogishima, Akira Uruno, Eiichi N. Kodama, Naoya Fujino, Tadahisa Numakura, Tomohiro Ichikawa, Ayumi Mitsune, Takashi Ohe, Kengo Kinoshita, Masakazu Ichinose, Hisatoshi Sugiura and Masayuki Yamamoto.

Supplementary Table 1 Baseline characteristics of the subjects for investigating genetic factors which determine lung function and FeNO in ToMMo study.

	CommCohort	BirThree Cohort
Number, Total	14,061	5,661
Number, male (%)	4,277 (30.4)	1,625 (28.7)
Median age, year (1st Qu. - 3rd Qu.)	67.01<br>(57.07 - 71.07)	39.07 (24.07)<br>(34.04 - 58.11)
Age range, year	22.1 - 90.0	20.1 - 88.1
Median Height, cm (1st Qu. - 3rd Qu.)	157.6<br>(152.5 - 163.4)	160.4<br>(155.6 - 166.6)
Number, never-smoker (%)	9460 (67.3)	3345 (59.1)
Number, ex-smoker (%)	3411 (24.3)	1536 (27.1)
Number, current smoker (%)	1190 (8.4)	780 (13.8)
Median FEV_1, L (1st Qu. - 3rd Qu.)	2.28<br>(1.95 – 2.68)	2.78<br>(2.41 - 3.21)
Median FVC, L (1st Qu. - 3rd Qu.)	2.83<br>(2.43 – 3.32)	3.30<br>(2.88 - 3.83)
Median FEV1/FVC, % (1st Qu. - 3rd Qu.)	80.79<br>(77.13 – 84.15)	83.69<br>(79.93 - 87.39)
Median FeNO, ppb (1st Qu. - 3rd Qu.)	19.00<br>(13.00 – 27.00)	19.00<br>(13.00 - 29.00)
Number, Asthma (%)	749 (5.3)	451 (8.0)
Number, COPD (%)	39 (0.3)	7 (0.12)

CommCohort, ToMMo Community-Based Cohort Study; BirThree Cohort, ToMMo Birth and Three-Generation Cohort Study GWAS, genome wide association study; Qu., quartile; FEV_1, Forced expiratory volume in 1 second; FVC, forced vital capacity; FeNO, fractional exhaled nitric oxide; ppb: parts-per-billion; COPD: chronic obstructive pulmonary disease.

Supplementary Table 2 Multiple regression analysis for FEV_1/FVC in both the Discovery and Validation cohorts.

Multiple linear regression analysis in the Discovery cohort
Objective variable: FEV_1/FVC
Explanatory variables: sex, age, FeNO, smoking status

	Estimate (β)	Estimate (B)	Std.Error	95%CI	t.value	p.value		VIF
Intercept	–	90.328434	0.330654	[89.68 , 90.977]	273.181	<2e-16	***	
sex	0.06567	0.839719	0.125371	[0.594 , 1.085]	6.698	2.19E-11	***	1.54
age	-0.2805	-0.146217	0.004271	[-0.155 , -0.138]	-34.236	<2e-16	***	1.075
FeNO	-0.08672	-0.031386	0.002893	[-0.037 , -0.026]	-10.849	<2e-16	***	1.024
smoking status	-0.12	-1.504479	0.119829	[-1.739 , -1.27]	-12.555	<2e-16	***	1.463
R-squared:	0.1227,	Adjusted.R-squared:	0.1224	AIC:	48002.88	BIC:	48040.64	
F-statistic:	491.3	on	4	and	14056	DF,	p-value:	<2.2e-16

Multiple linear regression analysis in the Validation cohort
Objective variable: FEV_1/FVC
Explanatory variables: sex, age, FeNO, smoking status

	Estimate (β)	Estimate (B)	Std.Error	95%CI	t.value	p.value		VIF
Intercept	-	91.133101	0.320481	[90.505 , 91.761]	284.363	<2e-16	***	
sex	9.49E-02	1.229998	0.169487	[0.898 , 1.562]	7.257	4.49E-13	***	1.205
age	-3.95E-01	-0.165584	0.005063	[-0.176 , -0.156]	-32.707	<2e-16	***	1.029
FeNO	-1.03E-01	-0.030288	0.003532	[-0.037 , -0.023]	-8.576	<2e-16	***	1.021
smoking status	-6.53E-02	-0.779075	0.153008	[-1.079 , -0.479]	-5.092	3.66E-07	***	1.16
R-squared:	0.1972,	Adjusted.R-squared:	0.1966	AIC:	18792.82	BIC:	18830.58	
F-statistic:	347.2	on	4	and	5656	DF,	p-value:	<2.2e-16

Note multiple linear regression analysis revealed that FeNO, age and sex, as well as smoking status, are independently related factors to FEV_1/FVC in both cohorts.

Supplementary Table 3 Loci associated with the measures of FEV_1/FVC without the adjustment of FeNO at Discovery Stage.

Pheno-type	Locus	Gene	Sentinel SNP	Function	Ref	Alt	EA	Impu-ted	Discovery stage EA freq.	Beta	SE	*P*	Validation stage EA freq.	Beta	SE	*P*	Veri-fied	Rep-orted
FEV_1/ FVC	6p21.32	*RNF5*	rs41315238	intronic	AAG	A	A	N	0.1513	0.1545	0.0156	7.9 E-24	0.1510	0.1241	0.0235	1.6 E-07	Tier 2	N
		AGER	rs2070600	exonic	C	T	T	N	0.1493	0.1552	0.0157	7.8 E-24	0.1493	0.1271	0.0237	9.4 E-08	Tier 2	Y
		PPT2-EGFL8	rs10947233	ncRNA_ intronic	G	T	T	Y	0.1509	0.1518	0.0156	5.4 E-23	0.1507	0.1267	0.0235	8.8 E-08	Tier 2	Y
	4q22.1	*FAM13A*	4:89834173_ GGAAGAA_ GGAA	intronic	GGA-AGAA	GGAA	GGAA	Y	0.3997	0.0649	0.0114	8.1 E-09	0.3982	0.0838	0.0176	1.7 E-06	Tier 2	Y
	2q37.3	*LINC01940 ~HDAC4*	rs36119524	Inter-genic	C	T	T	Y	0.2767	0.0839	0.0124	1.6 E-11	0.2846	0.0585	0.0188	2.0 E-03	N	Y
	1p21.2	*PLPPR4 ~LINC01708*	1:99809254_ TA_T	Inter-genic	TA	T	T	Y	0.0349	0.1796	0.0315	1.8 E-08	0.0355	-0.0235	0.0471	5.7 E-01	N	Y

Covariate adjustment with age, sex, and smoking status for FEV_1/FVC.

Ref, reference allele; Alt, alternative allele; EA, effect allele; EA freq., EA frequency; SE, standard error; ncRNA, non-coding RNA.

Supplementary Table 4 Loci associated with the measures of FEV_1 with the adjustment of FeNO at Discovery Stage.

									Discovery stage				Validation stage					
Pheno-type	Locus	Gene	Sentinel SNP	Function	Ref	Alt	EA	Impu-ted	EA freq.	Beta	SE	*P*	EA freq.	Beta	SE	*P*	Veri-fied	Rep-orted
FEV_1	4q31.21	*GYPA~HHIP-AS1*	rs13107665	Inter-genic	A	G	G	Y	0.6770	0.0457	0.0079	5.4 E-09	0.6841	0.0423	0.0117	2.7 E-04	N	N
	5p12	*FGF10*	5:44381582_A_AC	intronic	A	AC	AC	Y	0.1163	0.0670	0.0116	7.7 E-09	0.1191	0.0447	0.0169	7.8 E-03	N	Y

Covariate adjustment with age, sex, height, smoking status and FeNO for FEV_1.
Ref, reference allele; Alt, alternative allele; EA, effect allele; EA freq., EA frequency; SE, standard error; ncRNA, non-coding RNA.

Supplementary Table 5
MAF of AGER rs2070600 in our study chorts and other ethnic populations

Database	Ethnic group	Number of subjects	MAF
Discovery cohort in our study	Japnese	14,061	0.1493
Validation chort in our study	Japnese	5,661	0.1493
gnomeAD	East Asian (overall)	9912	0.2071
gnomeAD	Other East Asian	7154	0.2185
gnomeAD	Korean	1909	0.1671
gnomeAD	Japanese	69	0.1377
gnomeAD	European (Finnish)	12531	0.06795
gnomeAD	South Asian	15189	0.06557
gnomeAD	European (non-Finnish)	62927	0.04608
gnomeAD	Ashkenazi Jewish	5130	0.01062
gnomeAD	African/African-American	11734	0.009417
gnomeAD	Latino/Admixed American	17659	0.006682
NARD	Koreans	850	0.169
NARD	Japanese	396	0.168
NARD	Mongolians	384	0.105
NARD	Han Chinese	91	0.269
NARD	Hong Kong	58	0.328

gnomeAD: https://gnomad.broadinstitute.org/variant/rs2070600?dataset=gnomad_r2_1
NARD: https://nard.macrogen.com/

Supplementary Table 6
Genetic correlation between FeNO and other traits related to asthma or COPD

Trait1	Trait2	Genetic correlation	SE	P-value
FeNO	Asthma	0.1627	0.1061	0.1251
	COPD	0.0659	0.1439	0.6471
	Pollinosis	0.5162	0.2322	**0.0262**[*]
	Atopic dermatitis	-0.0231	0.1668	0.8899
	Drug eruption	0.2159	0.3393	0.5245
	Eosinophil count	0.3656	0.1166	**0.0017**[**]
	BMI	0.0691	0.0674	0.3059
	FEV_1/FVC	-0.1424	0.1448	0.3255
	FEV_1	-0.0182	0.1285	0.8871
Asthma	COPD	0.4759	0.1008	**2.3614×10^{-6}**[**]
	Pollinosis	0.1557	0.1444	0.281
	Atopic dermatitis	0.0277	0.0904	0.7589
	Drug eruption	0.3788	0.2339	0.1052
	Eosinophil count	0.3927	0.0846	**3.4395×10^{-6}**[**]
	BMI	0.1046	0.0437	**0.0167**[*]
	FEV1/FVC	-0.2911	0.1094	**0.0078**[*]
	FEV_1	-0.1896	0.0966	**0.0496**[*]
COPD	Pollinosis	0.0545	0.1737	0.7537
	Atopic dermatitis	0.1405	0.2312	0.5435
	Drug eruption	0.1183	0.2719	0.6636
	Eosinophil count	0.1102	0.09	0.221
	BMI	-0.0695	0.059	0.2387
	FEV1/FVC	-0.4149	0.1385	**0.0027**[**]
	FEV_1	-0.0326	0.1363	0.8112
Pollinosis	Atopic dermatitis	0.1531	0.2289	0.5034
	Drug eruption	-0.0866	0.3746	0.8171
	Eosinophil count	-0.0936	0.136	0.4912
	BMI	-0.2227	0.0851	**0.0088**[*]
	FEV1/FVC	0.1035	0.1758	0.5561
	FEV_1	0.0372	0.1571	0.8129
Atopic dermatitis	Drug eruption	-0.0384	0.3715	0.9177
	Eosinophil count	0.2981	0.1157	**0.0099**[*]
	BMI	-0.0663	0.0748	0.3752
	FEV1/FVC	0.0221	0.1702	0.8965
	FEV_1	0.0922	0.1128	0.4138
Drug eruption	Eosinophil count	0.0408	0.2215	0.854
	BMI	0.0884	0.1085	0.4153
	FEV1/FVC	-0.2224	0.2742	0.4172
	FEV_1	-0.3479	0.2689	0.1956
Eosinophil count	BMI	0.0428	0.0439	0.3289
	FEV1/FVC	-0.0395	0.1139	0.7289
	FEV_1	-0.1471	0.0908	0.1053
BMI	FEV1/FVC	0.0455	0.061	0.4563
	FEV_1	0.0711	0.0491	0.1479
FEV1/FVC	FEV_1	0.5428	0.1028	**1.2853×10^{-7}**[**]

BMI, body mass index; FEV_1, forced expiratory volume in one second
FVC, forced vital capacity.
[*], nominal ($P < 0.05$) significance
[**], significance after Bonferroni correction for the number of pairwise comparisons

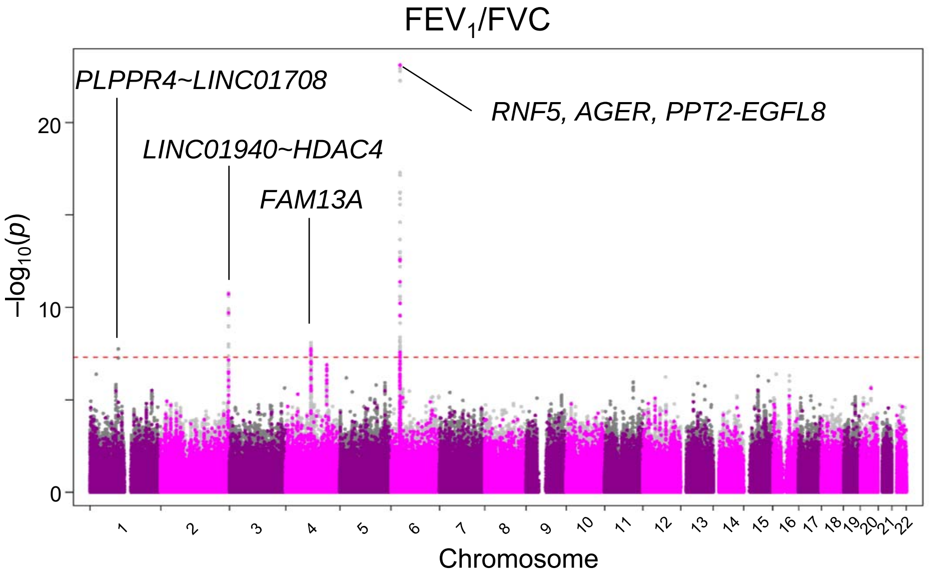

Supplementary Fig. 1 GWAS for association with the measures of FEV_1/FVC without the adjustment of FeNO at Discovery Stage. The Manhattan plots of GWAS for association with FEV_1/FVC ratio shows chromosomal position of variants exceeding the genome-wide significance threshold ($P < 5 \times 10^{-8}$ as indicated by the dotted red line). Gene names in black correspond to genetic loci which had $P < 5 \times 10^{-8}$ in Discovery Stage GWAS without the adjustment of FeNO. Colored circles mean the variants directly analyzed. Gray circles mean the variants detected by genotype imputation. GWAS, genome-wide association study; FEV_1, forced expiratory volume in one second; FVC, forced vital capacity.

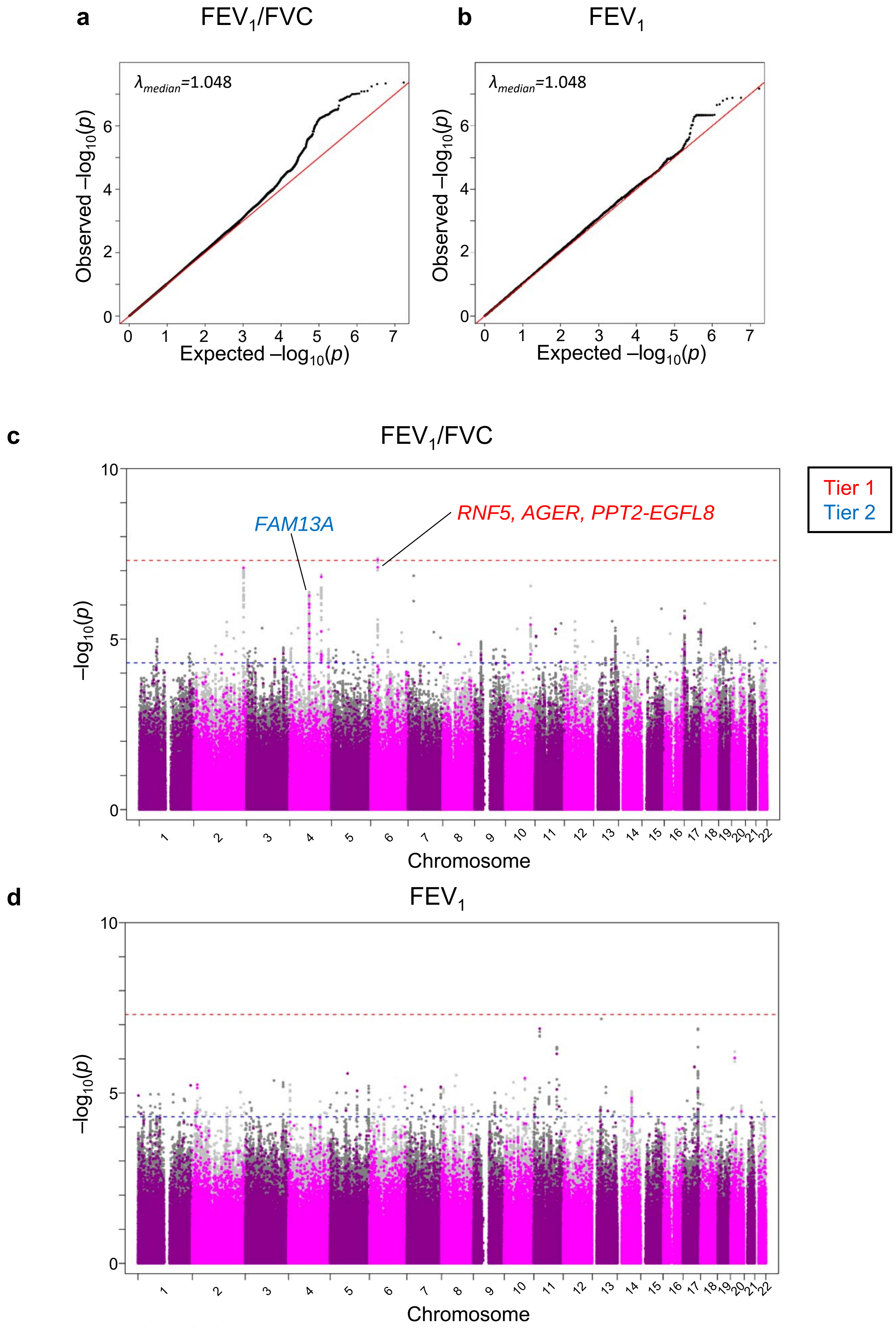

Supplementary Fig. 2 GWAS for association with the measures of lung function at Validation Stage. Quantile-quantile (QQ) plots of observed versus expected log10(P) values in FEV_1/FVC ratio (a) and FEV_1 (b) are shown with the inflation factor (λ). The Manhattan plots of GWAS for association with FEV_1/FVC ratio (c) and FEV_1 (d) shows chromosomal position of variants exceeding the genome-wide significance threshold ($P < 5 \times 10^{-8}$ as indicated by the dotted red line). Gene names in red correspond to tier 1 loci ($P < 5 \times 10^{-8}$ both in Discovery Stage and Validation Stage); gene names in blue correspond to tier 2 loci ($P < 5 \times 10^{-8}$ in Discovery Stage and $P < 5 \times 10^{-5}$ (indicated by the dotted blue line) in Validation Stage with consistent directions of effect). Colored circles mean the variants directly analyzed. Gray circles mean the variants detected by genotype imputation. GWAS, genome-wide association study; FEV_1, forced expiratory volume in one second; FVC, forced vital capacity.

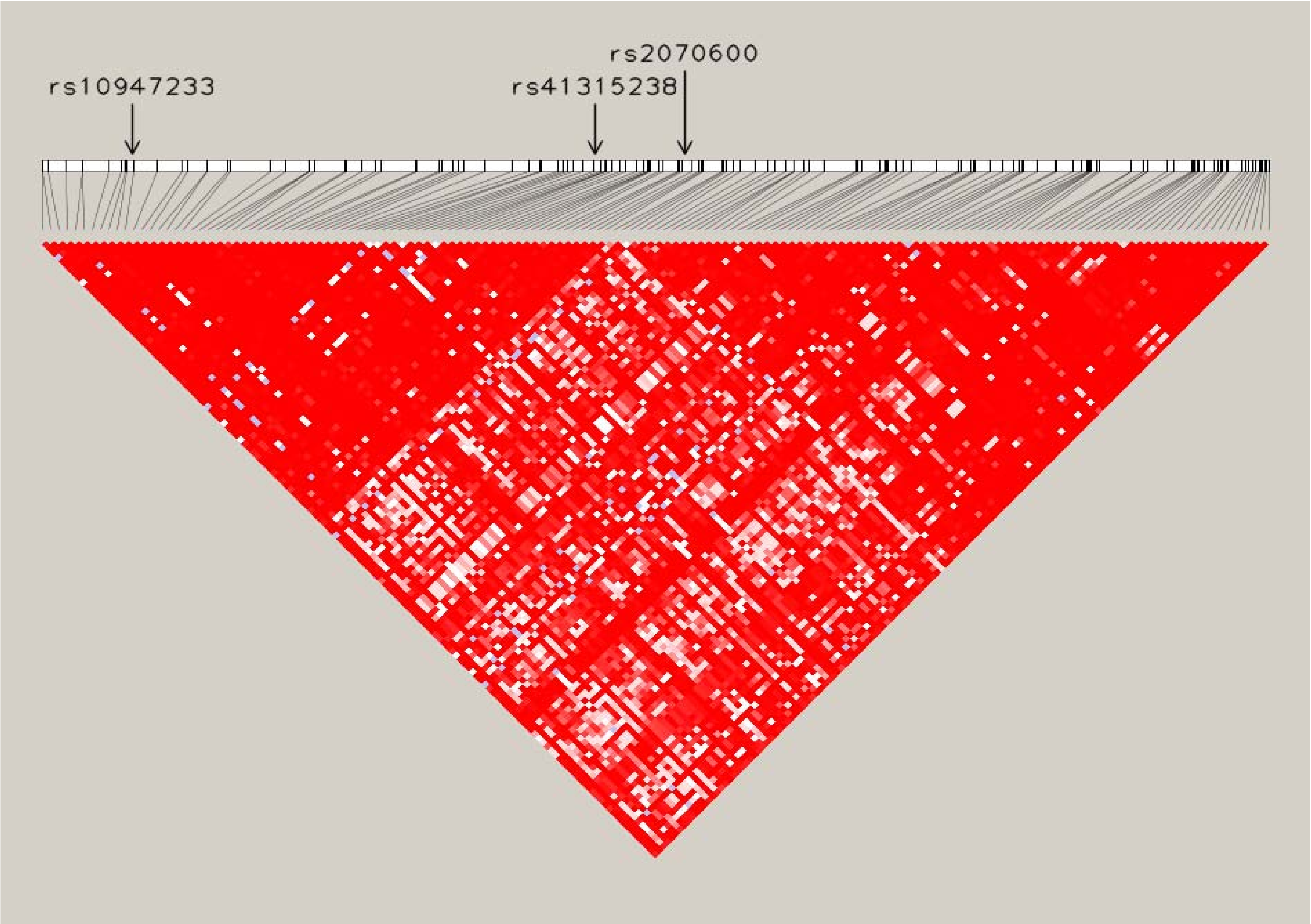

SNP1	SNP2	r^2	D'
rs10947233	rs41315238	0.994686	0.997479
rs10947233	rs2070600	0.981553	0.997446
rs41315238	rs2070600	0.983952	0.998596

Supplementary Fig. 3 An linkage disequilibrium (LD) plot based on r^2 for chr6:32120000-32180000, a neighboring region of sentinel SNPs in chr6, rs10947233 in *PPT2-EGFL8,* rs41315238 in *RNF5, and* rs2070600 in *AGER*. Note these three SNPs are in the same strong LD block.

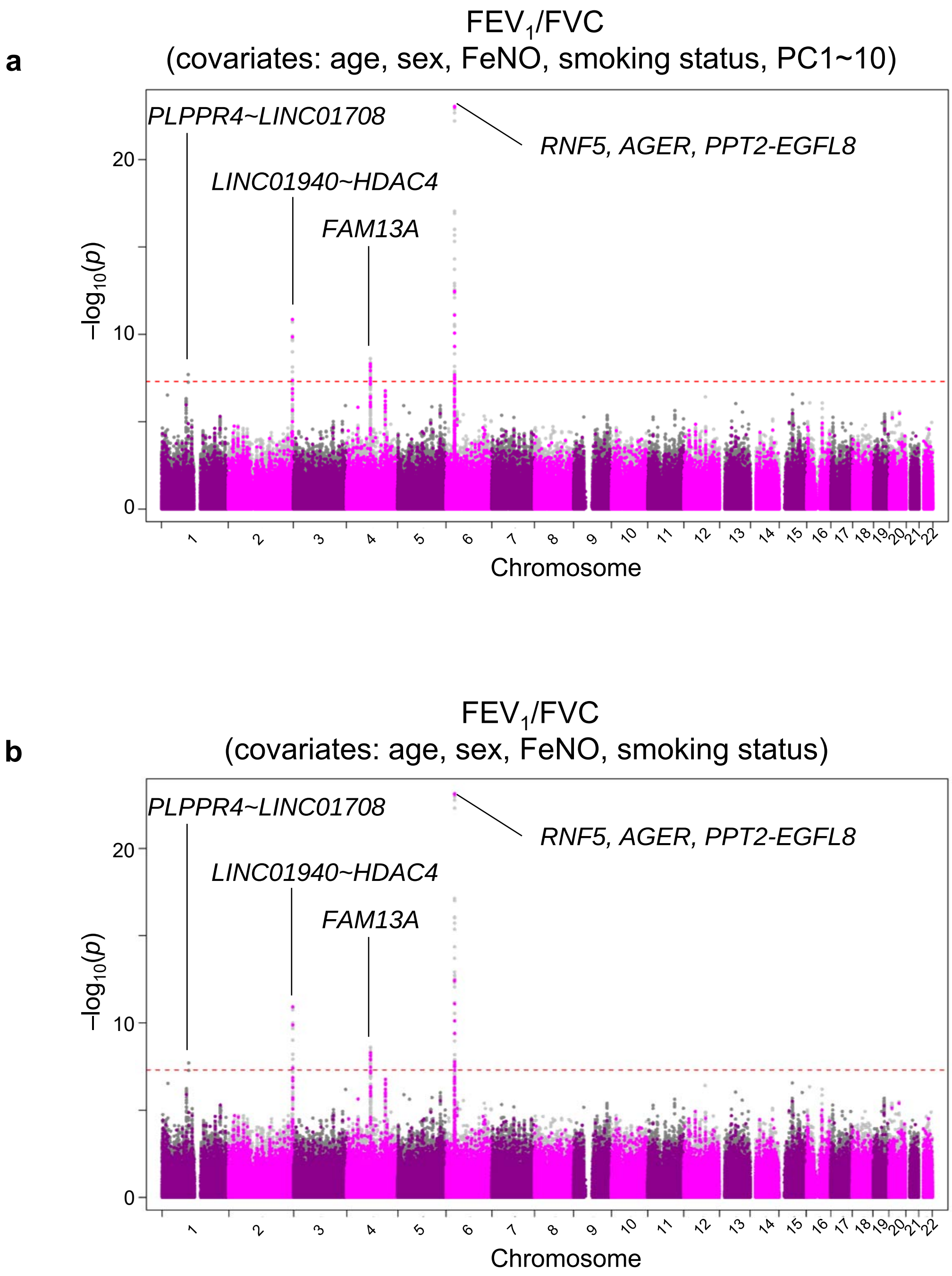

Supplementary Fig. 4 Comparison of GWAS for FEV_1/FVC between with and without the adjustment of the principal components in the Discovery cohort. The Manhattan plots of GWAS for FEV_1/FVC with (a) or without (b) the adjustment of the principal components (PC1~10) shows chromosomal position of variants exceeding the genome-wide significance threshold ($P < 5 \times 10^{-8}$ as indicated by the dotted red line). Gene names correspond to genetic loci which had $P < 5 \times 10^{-8}$ in Discovery Stage GWAS. Colored circles mean the variants directly analyzed. Gray circles mean the variants detected by genotype imputation. GWAS, genome-wide association study; FEV_1, forced expiratory volume in one second; FVC, forced vital capacity.

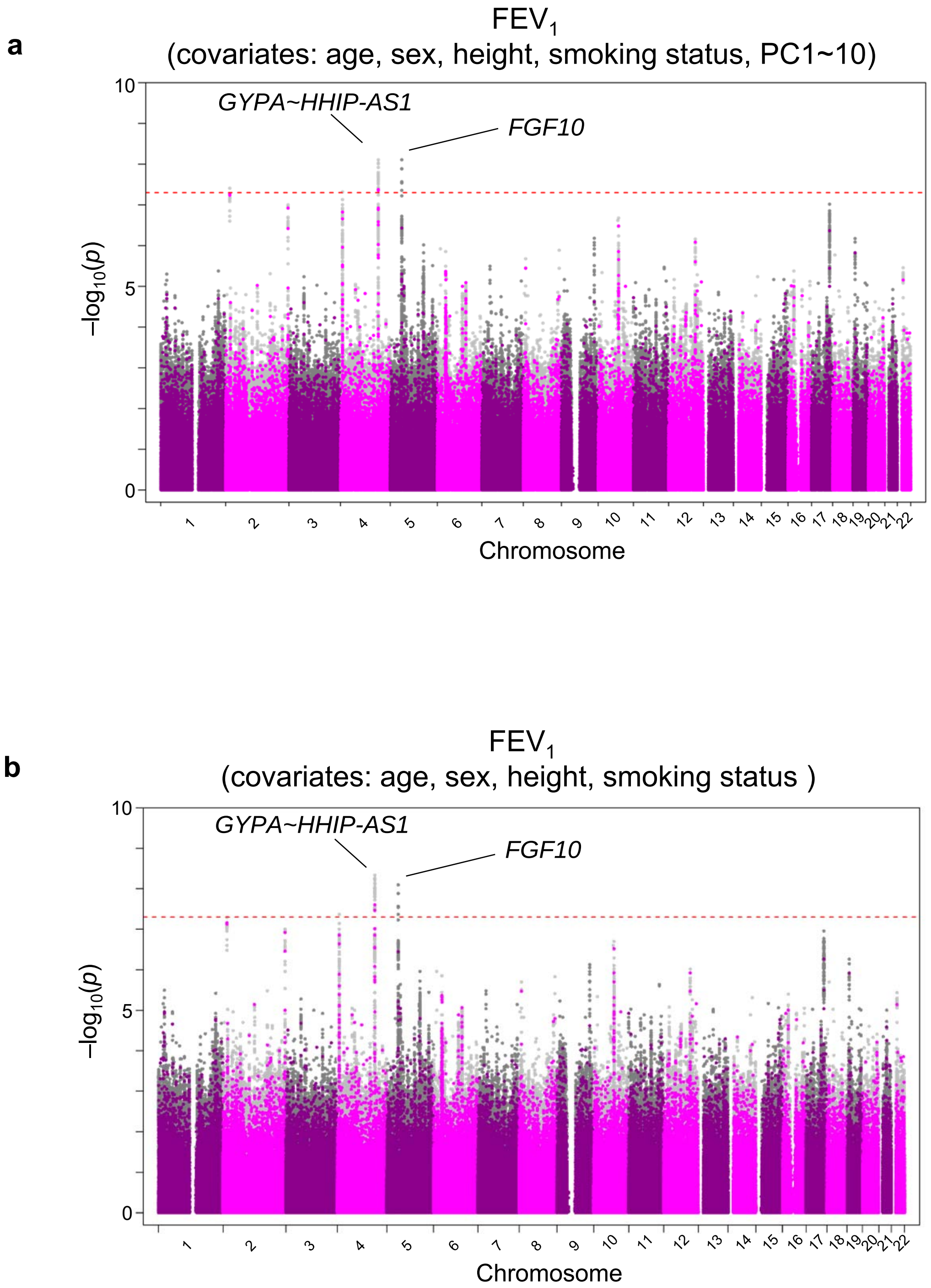

Supplementary Fig. 5 Comparison of GWAS for FEV_1 between with and without the adjustment of the principal components in the Discovery cohort. The Manhattan plots of GWAS for FEV_1 with (a) or without (b) the adjustment of the principal components (PC1~10) shows chromosomal position of variants exceeding the genome-wide significance threshold ($P < 5 \times 10^{-8}$ as indicated by the dotted red line). Gene names in black correspond to genetic loci which had $P < 5 \times 10^{-8}$ in Discovery Stage GWAS. Colored circles mean the variants directly analyzed. Gray circles mean the variants detected by genotype imputation. GWAS, genome-wide association study; FEV_1, forced expiratory volume in one second.

a

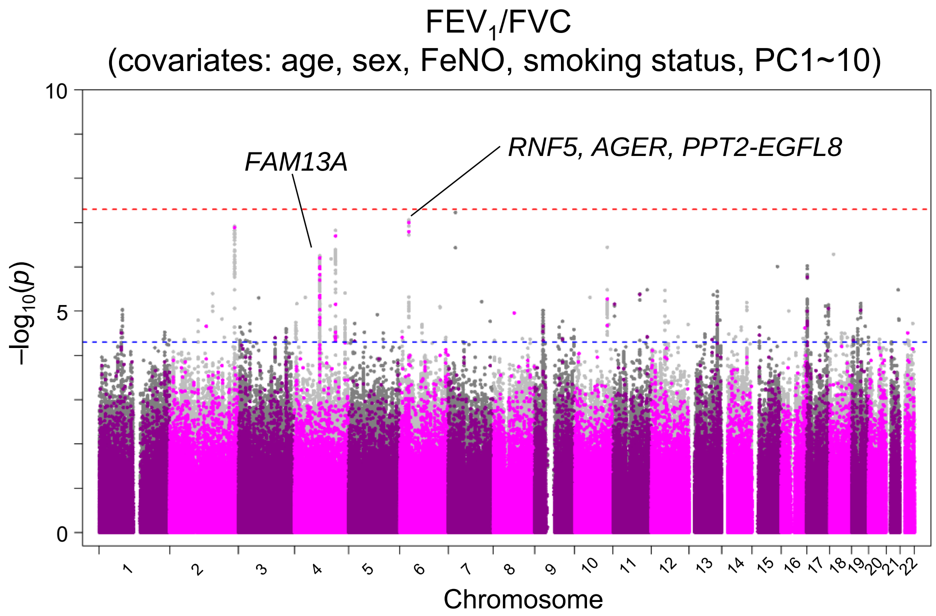

b

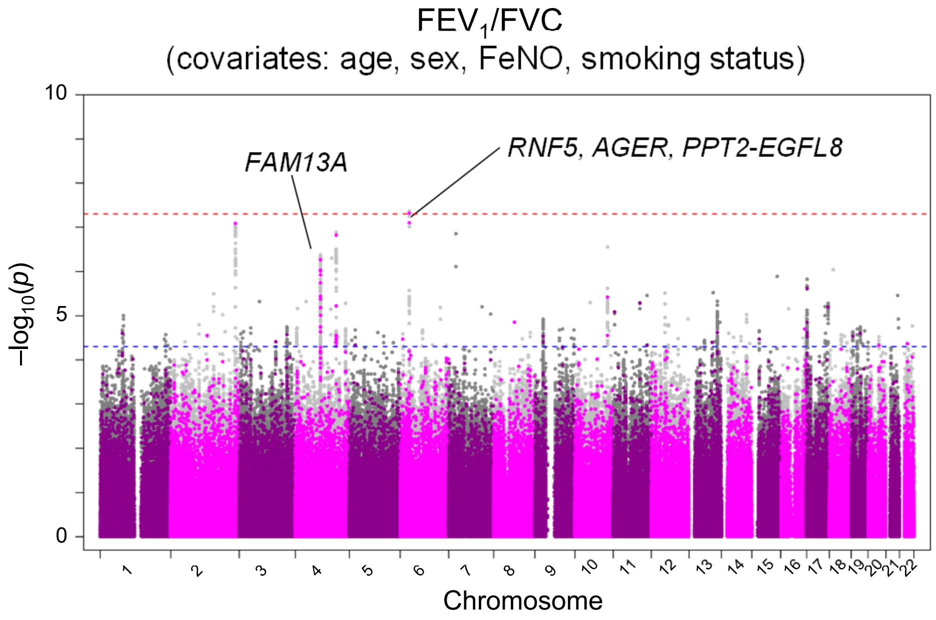

Supplementary Fig. 6 Comparison of GWAS for FEV$_1$/FVC between with and without the adjustment of the principal components in the Validation cohort. The Manhattan plots of GWAS for FEV$_1$/FVC with (a) or without (b) the adjustment of the principal components (PC1~10) shows chromosomal position of variants. The dotted red line indicates the genome-wide significance threshold ($P < 5 \times 10^{-8}$). The dotted blue line indicates the genome-wide suggestive level ($P < 5 \times 10^{-5}$). Gene names correspond to loci whose $P < 5 \times 10^{-8}$ in the Discovery Stage and $P < 5 \times 10^{-5}$ in the Validation Stage. Colored circles mean the variants directly analyzed. Gray circles mean the variants detected by genotype imputation. GWAS, genome-wide association study; FEV$_1$, forced expiratory volume in one second; FVC, forced vital capacity.

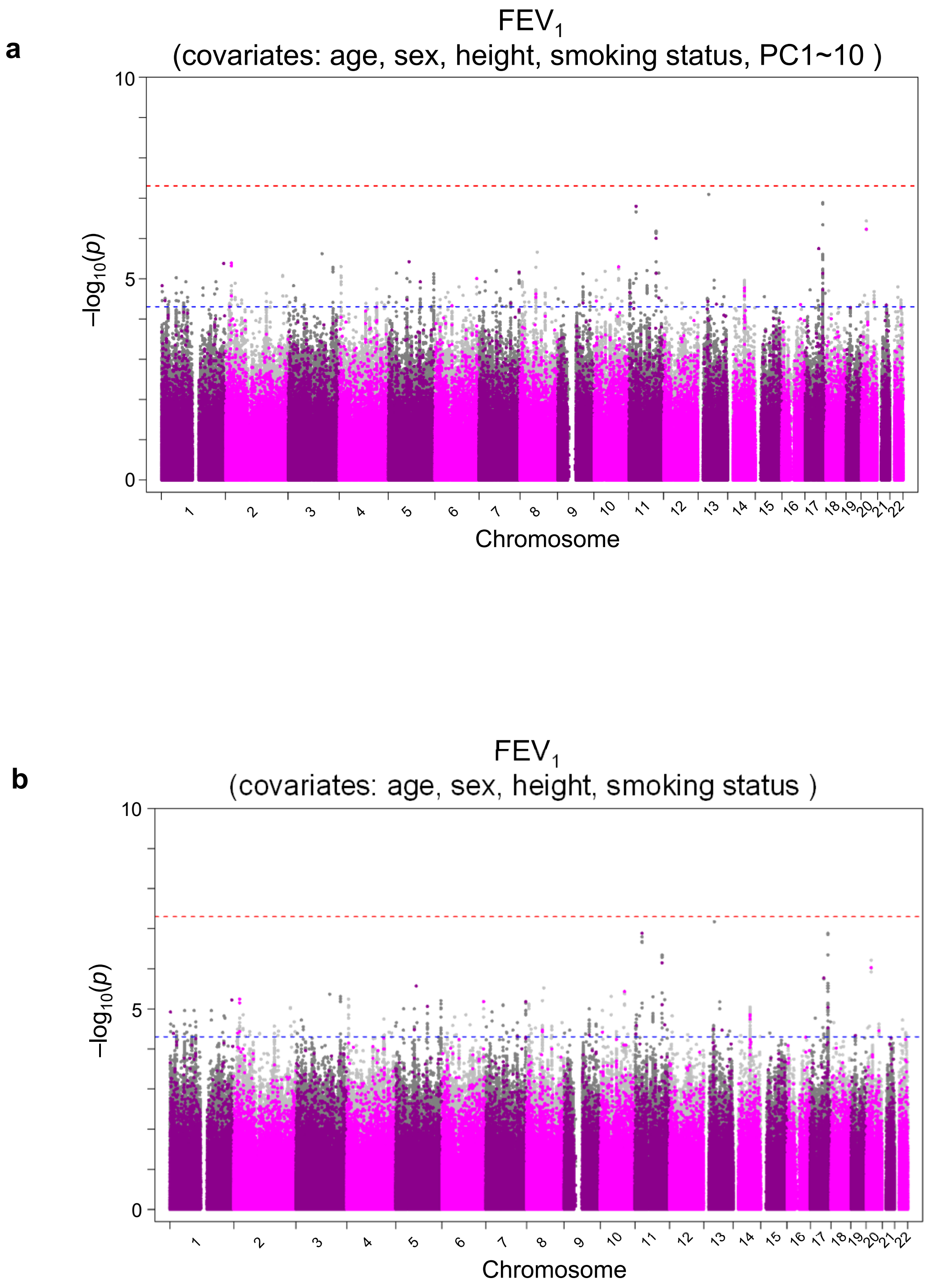

Supplementary Fig. 7 Comparison of GWAS for FEV_1 between with and without the adjustment of the principal components in the Validation cohort. The Manhattan plots of GWAS for FEV_1 with (a) or without (b) the adjustment of the principal components (PC1~10) shows chromosomal position of variants. The dotted red line indicates the genome-wide significance threshold ($P < 5 \times 10^{-8}$). The dotted blue line indicates the genome-wide suggestive level ($P < 5 \times 10^{-5}$). Colored circles mean the variants directly analyzed. Gray circles mean the variants detected by genotype imputation. GWAS, genome-wide association study; FEV_1, forced expiratory volume in one second.

a

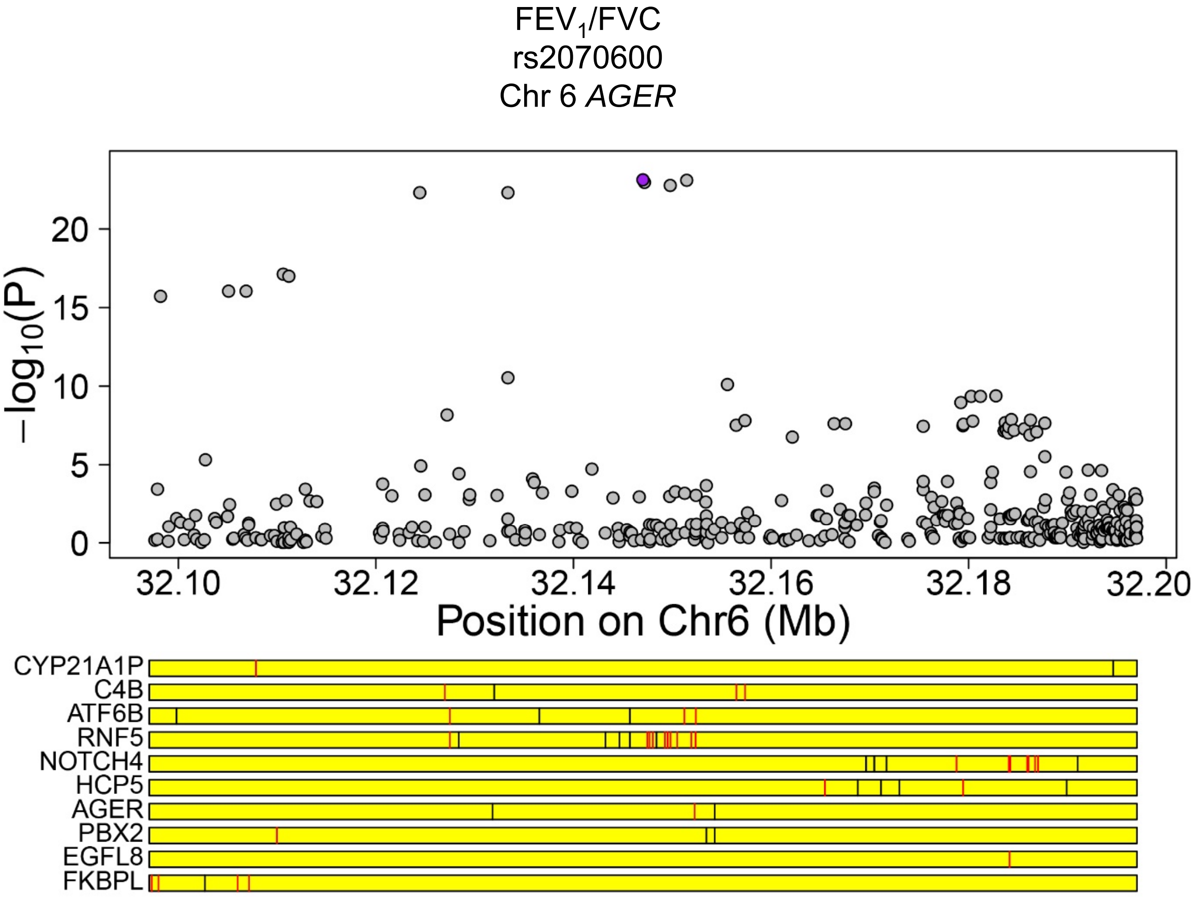

b

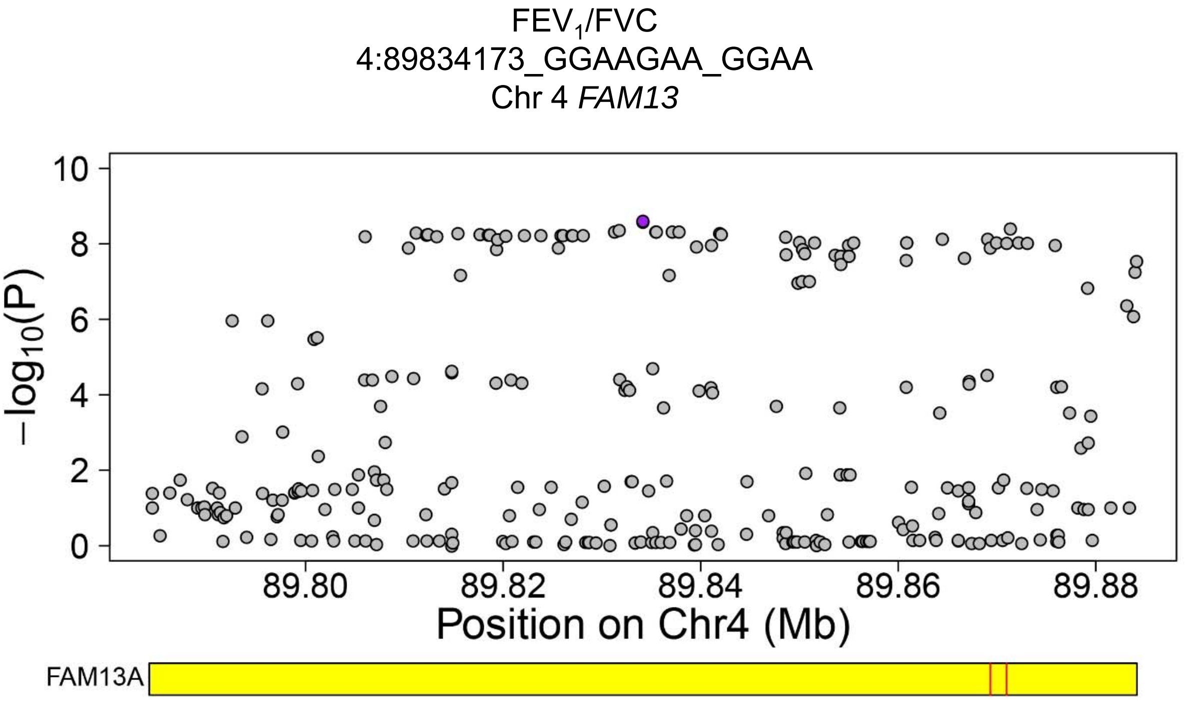

Supplementary Fig. 8 Regional plots of the validated signals of GWAS for FEV_1/FVC with genes whose expression changes were possibly caused by the corresponding signals. (a-b) Dots in the plots indicate variants around each signal and their minus log 10 p-values, and a dot in purple each plot indicates the top variant of the signals in the Discovery stage alone. Vertical bars for each gene indicate physical positions of highly confident eQTL associated variants. Red vertical bars indicate physical positions of the eQTL associated variants located within 1 kb distance from one of genome-wide significant variants while black vertical bars indicate physical position of others.

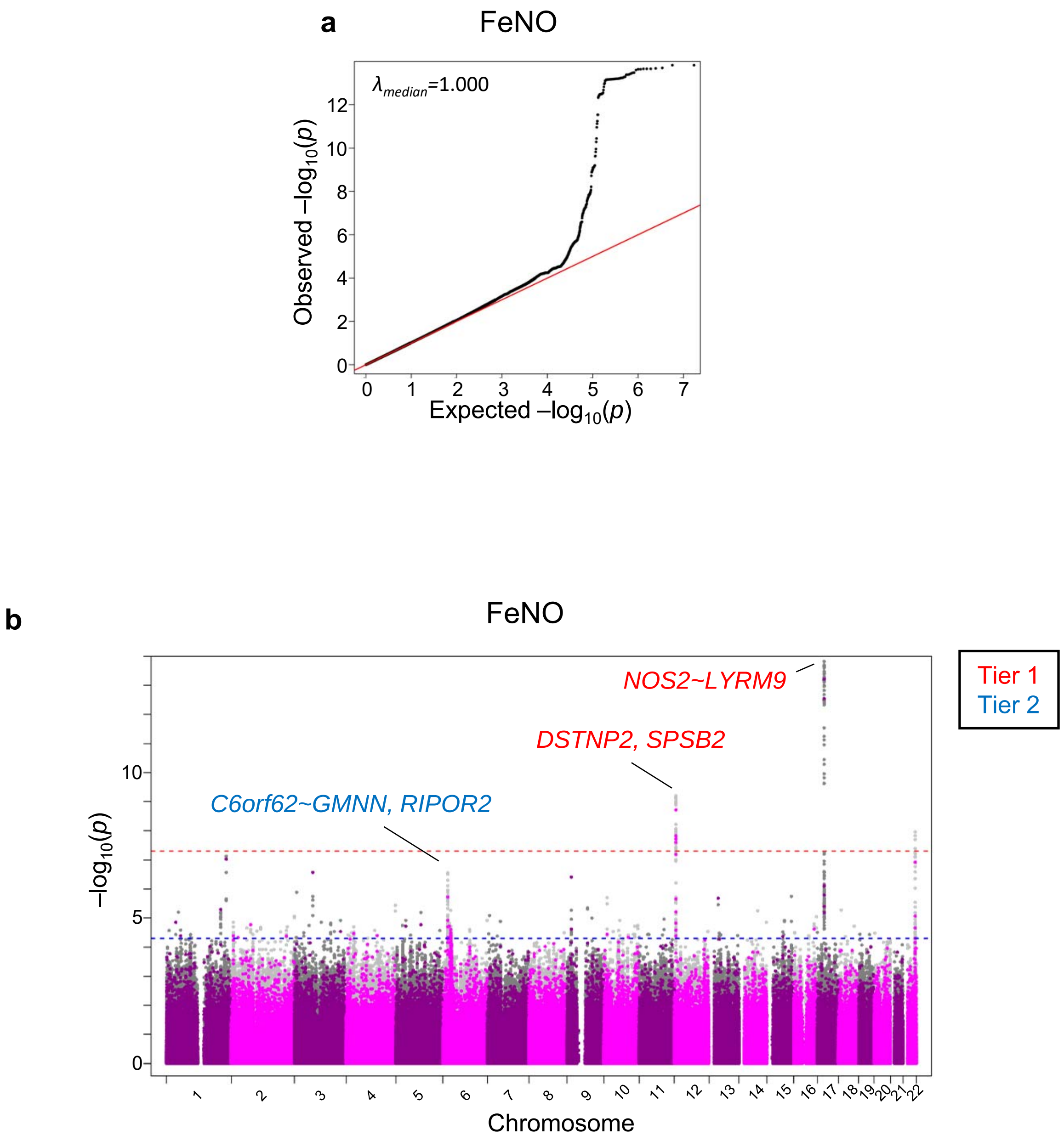

Supplementary Fig. 9 GWAS for association with the levels of FeNO Validation Stage. A Quantile-quantile (QQ) plot of observed versus expected log10(*P*) values in FeNO is shown with the inflation factor (λ). (b)The Manhattan plot of GWAS for association with FeNO shows chromosomal position of variants exceeding the genome-wide significance threshold ($P < 5 \times 10^{-8}$ as indicated by the dotted red line). Gene names in red correspond to tier 1 loci ($P < 5 \times 10^{-8}$ both in Discovery Stage and Validation Stage); gene names in blue correspond to tier 2 loci ($P < 5 \times 10^{-8}$ in Discovery Stage and $P < 5 \times 10^{-5}$ (indicated by the dotted blue line) in Validation Stage with consistent directions of effect). Colored circles mean the variants directly analyzed. Gray circles mean the variants detected by genotype imputation. GWAS, genome-wide association study; FeNO, exhaled nitric oxide.

a

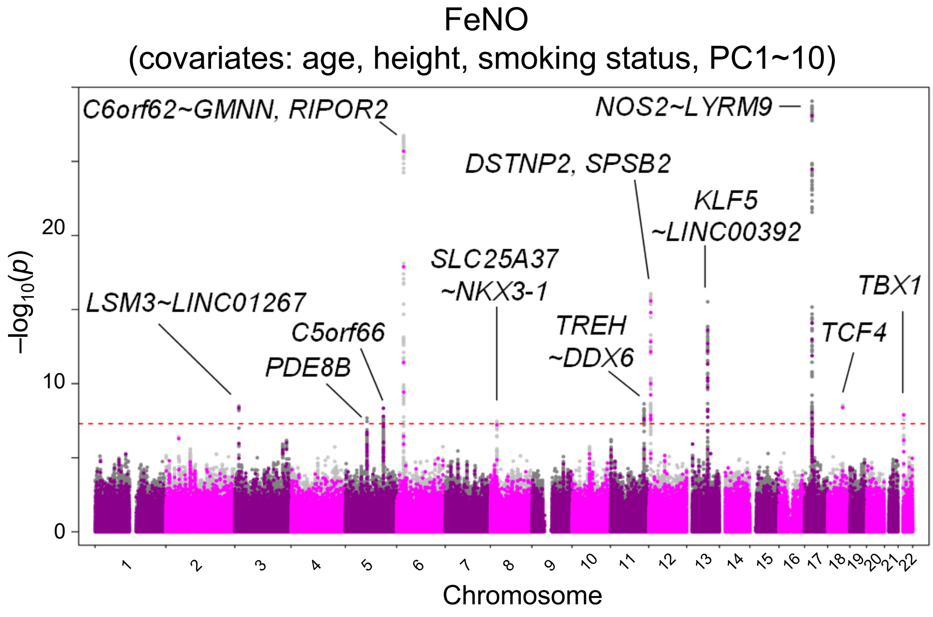

b

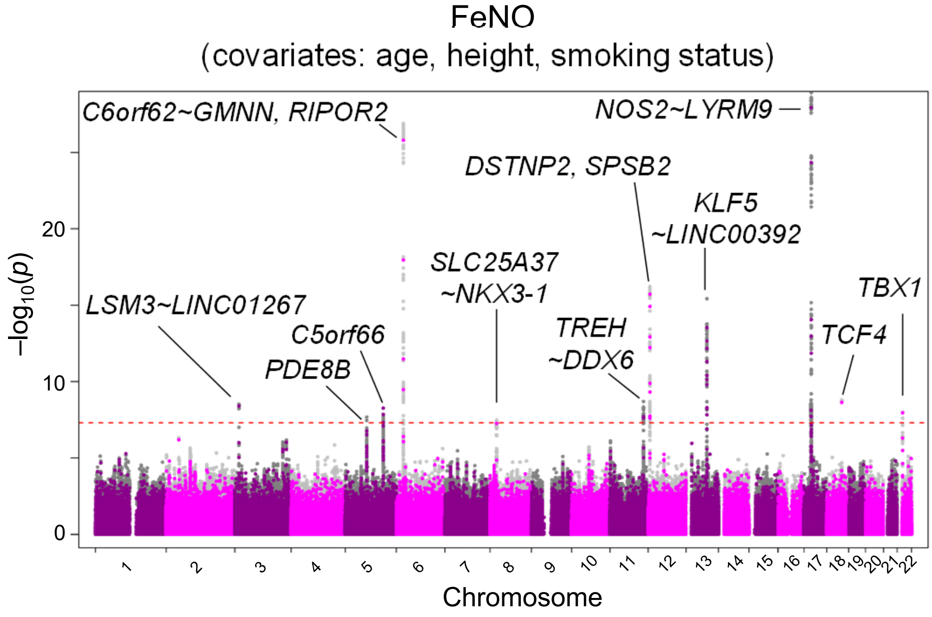

Supplementary Fig. 10 Comparison of GWAS for FeNO between with and without the adjustment of the principal components in the Discovery cohort. The Manhattan plots of GWAS for FeNO with (a) or without (b) the adjustment of the principal components (PC1~10) shows chromosomal position of variants exceeding the genome-wide significance threshold ($P < 5 \times 10^{-8}$ as indicated by the dotted red line). Gene names in black correspond to genetic loci which had $P < 5 \times 10^{-8}$ in Discovery Stage GWAS. Colored circles mean the variants directly analyzed. Gray circles mean the variants detected by genotype imputation. GWAS, genome-wide association study; FeNO, exhaled nitric oxide.

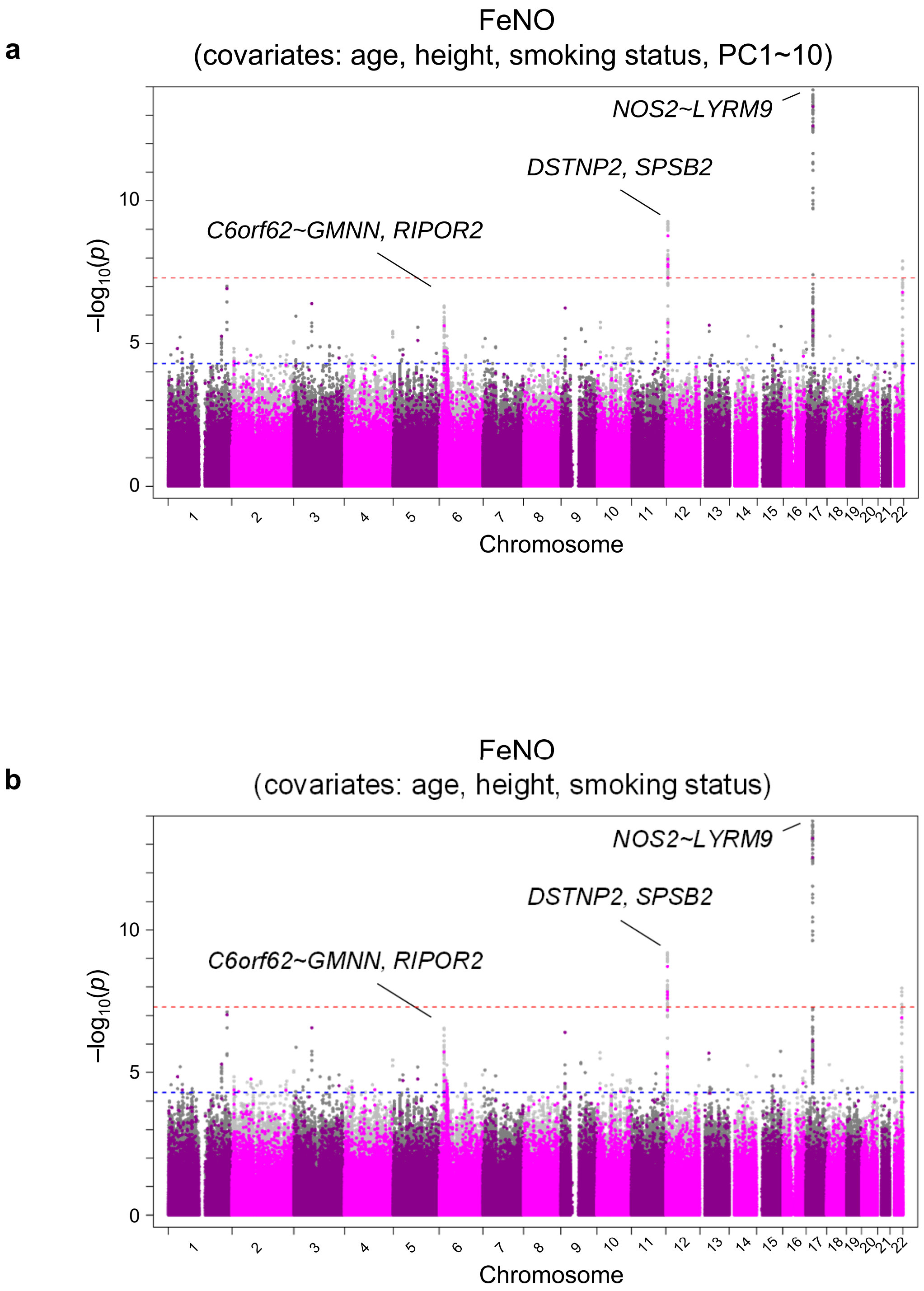

Supplementary Fig. 11 Comparison of GWAS for FeNO between with and without the adjustment of the principal components in the Validation cohort. The Manhattan plots of GWAS for FeNO with (a) or without (b) the adjustment of the principal components (PC1~10) shows chromosomal position of variants. The dotted red line indicates the genome-wide significance threshold ($P < 5 \times 10^{-8}$). The dotted blue line indicates the genome-wide suggestive level ($P < 5 \times 10^{-5}$). Gene names correspond to loci whose $P < 5 \times 10^{-8}$ in the Discovery Stage and $P < 5 \times 10^{-5}$ in the Validation Stage. Colored circles mean the variants directly analyzed. Gray circles mean the variants detected by genotype imputation. GWAS, genome-wide association study; FeNO, exhaled nitric oxide.

a

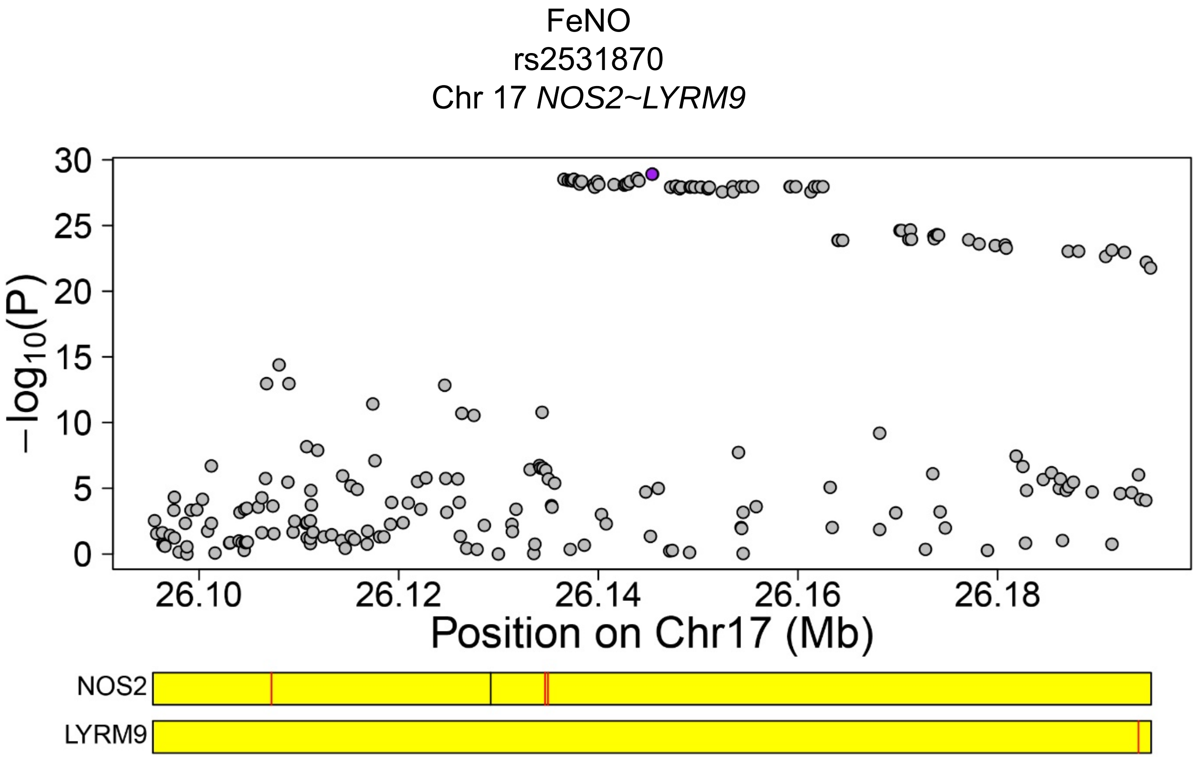
FeNO
rs2531870
Chr 17 NOS2~LYRM9
−log10(P)
Position on Chr17 (Mb)
NOS2
LYRM9

b

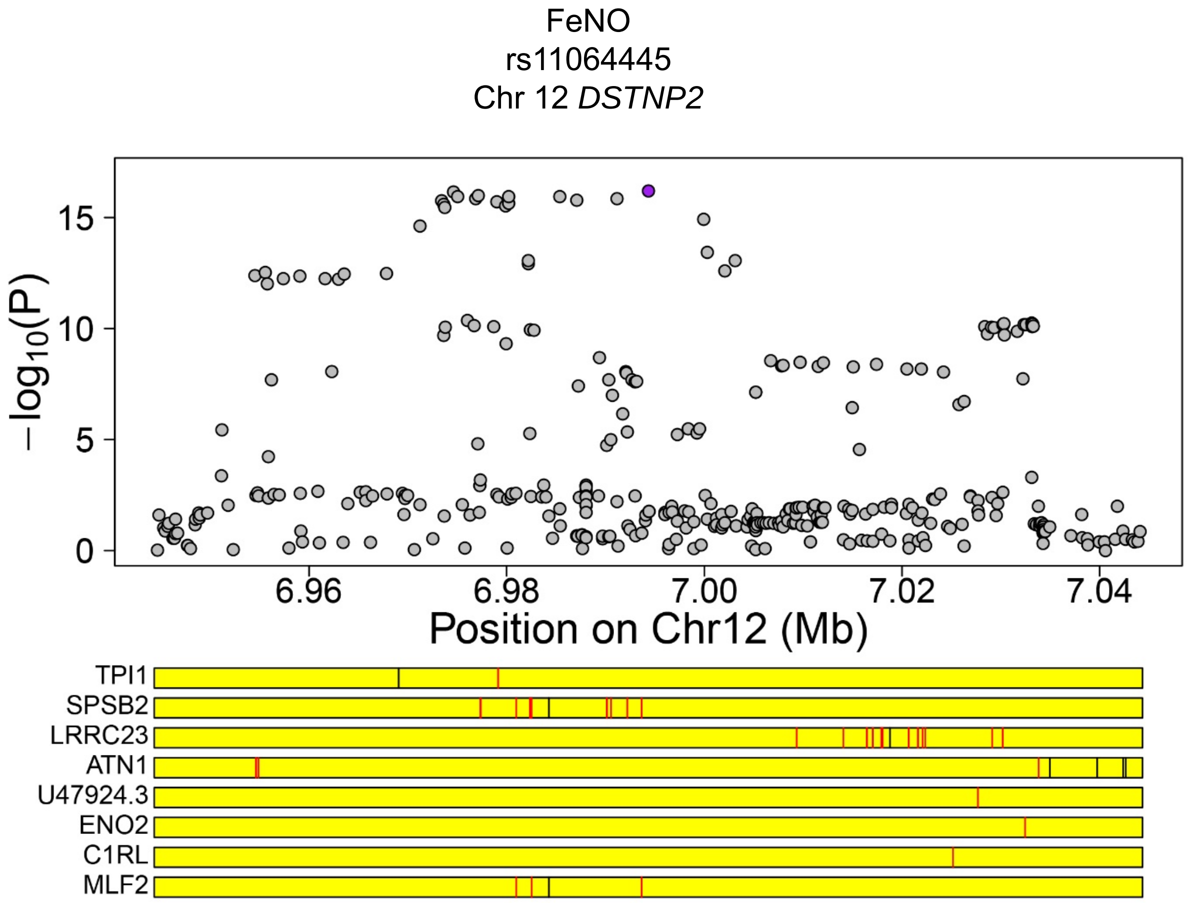
FeNO
rs11064445
Chr 12 DSTNP2
−log10(P)
Position on Chr12 (Mb)
TPI1
SPSB2
LRRC23
ATN1
U47924.3
ENO2
C1RL
MLF2

c

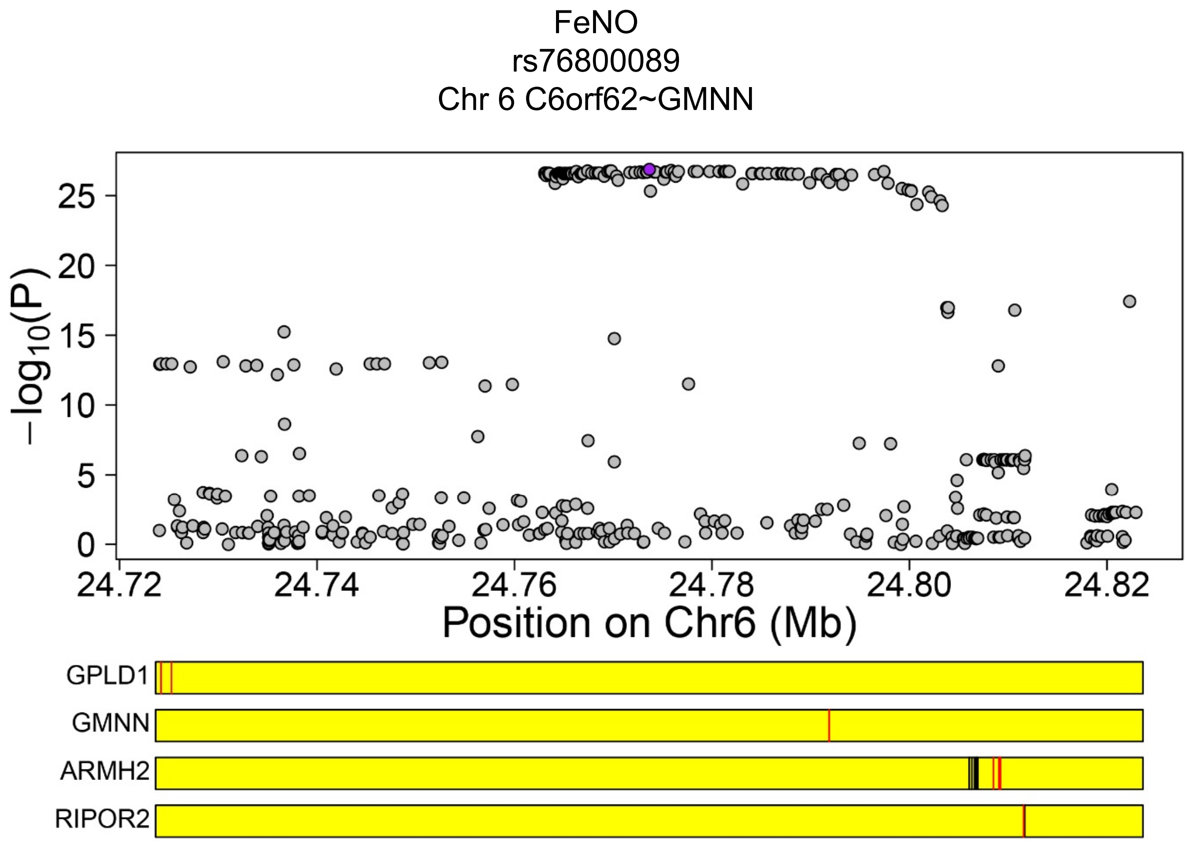

Supplementary Fig. 12 Regional plots of the validated signals of GWAS for FeNO with genes whose expression changes were possibly caused by the corresponding signals. (a-c) Dots in the plots indicate variants around each signal and their minus log 10 p-values, and a dot in purple each plot indicates the top variant of the signals. Vertical bars for each gene indicate physical positions of highly confident eQTL associated variants. Red vertical bars indicate physical positions of the eQTL associated variants located within 1 kb distance from one of genome-wide significant variants while black vertical bars indicate physical position of others.

a

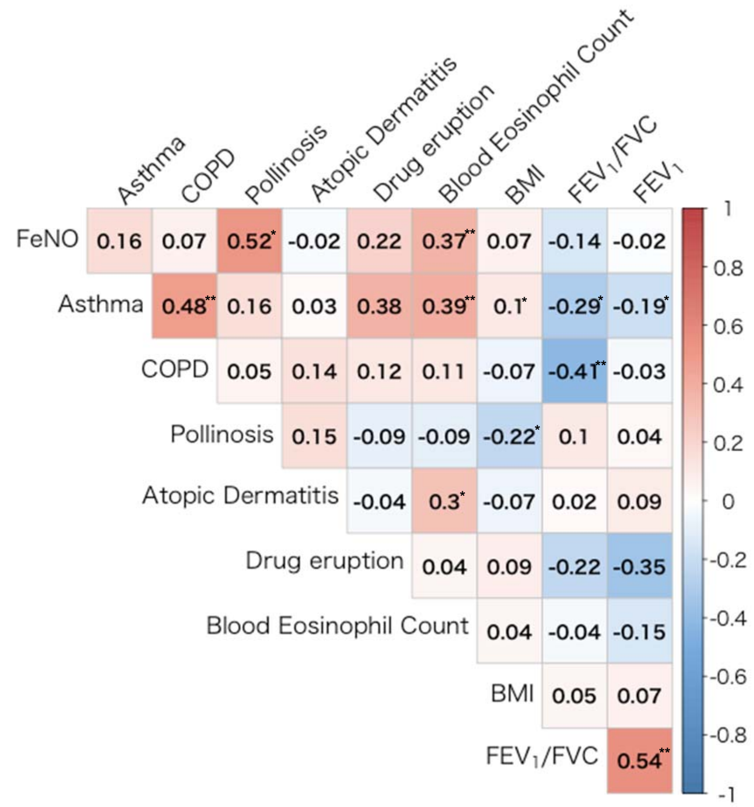

b

Locus	3p25.1	5q13.3	5q31.1	6p22.3	8p21.2	11q23.3	12p13.31	13q22.1	17q11.2	18q21.2	22q11.21
Gene	*LSM3*	*PDE8B*	*C5orf66*	*RIPOR2*	*NKX3-1*	*CXCR5*	*SPSB2*	*KLF5*	*NOS2*	*TCF4*	*TBX1*
Asthma				■		■					
Allergic rhinitis			■					■			
Allegic dermatitis								■			
Eosinophil counts		■			■	■			■		
Post bronchodilator FEV1/FVC ratio				■						■	
FEV1			■								
genes which has the function associated with NO production							■		■		

Supplementary Fig. 13 Genetic correlation of FeNO with asthma-related traits. (a) The genetic correlation (from LD score regression) between FeNO and other traits including asthma-related traits. An asterisk indicates nominal (P < 0.05) significance. A double asterisk indicates significance after Bonferroni correction for the number of pairwise comparisons. Asthma, COPD, pollinosis, atopic dermatitis, drug eruption, eosinophil count, and BMI data were obtained from BioBank Japan[46-48]. FeNO, FEV_1/FVC, and FEV_1 data were derived from this our study. (b) Overlap of genome-wide-significant-loci for FeNO and selected traits from the GWAS Catalog[49]. r_g, genetic correlation; FeNO, exhaled nitric oxide; BMI, body mass index.
