# Supplementary material for: Genetic loci for lung function in Japanese adults with adjustment for exhaled nitric oxide levels as airway inflammation indicator
Source: Commun Biol. 2021 Nov 15;4:1288. doi: 10.1038/s42003-021-02813-8 (PMC8593164; doi:10.1038/s42003-021-02813-8)
Supplement: Supplementary file 3 — Description of Supplementary Data Files [file 42003_2021_2813_MOESM3_ESM.pdf]

Description of Supplementary Data

File name: Supplementary Data 1
Description: Variants associated with FEV_1/FVC at Discovery Stage.

File name: Supplementary Data 2
Description: Variants associated with FEV_1 at Discovery Stage.

File name: Supplementary Data 3
Description: Variants associated with FEV_1/FVC at Discovery Stage and validated at Validation Stage.

File name: Supplementary Data 4
Description: Variants associated with FeNO at Discovery Stage.

File name: Supplementary Data 5
Description: Variants associated with FENO at Discovery Stage and validated at Validation Stage.

File name: Supplementary Data 6
Description: Source data underlying boxplots shown in the Main Figures (Fig. 4a, 4b, 8a, 8b, 8c).
