# Supplementary material for: Genetic loci for lung function in Japanese adults with adjustment for exhaled nitric oxide levels as airway inflammation indicator
Source: Commun Biol. 2021 Nov 15;4:1288. doi: 10.1038/s42003-021-02813-8 (PMC8593164; doi:10.1038/s42003-021-02813-8)
Supplement: Supplementary file 10 — Reporting Summary [file 42003_2021_2813_MOESM10_ESM.pdf]

nature portfolio

Corresponding author(s): Masayuki Yamamoto

Last updated by author(s): Oct 17, 2021

Reporting Summary

Nature Portfolio wishes to improve the reproducibility of the work that we publish. This form provides structure for consistency and transparency in reporting. For further information on Nature Portfolio policies, see our Editorial Policies and the Editorial Policy Checklist.

Statistics

For all statistical analyses, confirm that the following items are present in the figure legend, table legend, main text, or Methods section.

n/a	Confirmed	
☐	☒	The exact sample size (n) for each experimental group/condition, given as a discrete number and unit of measurement
☐	☒	A statement on whether measurements were taken from distinct samples or whether the same sample was measured repeatedly
☐	☒	The statistical test(s) used AND whether they are one- or two-sided<br>*Only common tests should be described solely by name; describe more complex techniques in the Methods section.*
☐	☒	A description of all covariates tested
☐	☒	A description of any assumptions or corrections, such as tests of normality and adjustment for multiple comparisons
☐	☒	A full description of the statistical parameters including central tendency (e.g. means) or other basic estimates (e.g. regression coefficient) AND variation (e.g. standard deviation) or associated estimates of uncertainty (e.g. confidence intervals)
☐	☒	For null hypothesis testing, the test statistic (e.g. F, t, r) with confidence intervals, effect sizes, degrees of freedom and P value noted<br>*Give P values as exact values whenever suitable.*
☒	☐	For Bayesian analysis, information on the choice of priors and Markov chain Monte Carlo settings
☒	☐	For hierarchical and complex designs, identification of the appropriate level for tests and full reporting of outcomes
☐	☒	Estimates of effect sizes (e.g. Cohen's d, Pearson's r), indicating how they were calculated

Our web collection on statistics for biologists contains articles on many of the points above.

Software and code

Policy information about availability of computer code

Data collection	We fully described in the Methods.
Data analysis	ALL software used in our study are shown in the Methods.

For manuscripts utilizing custom algorithms or software that are central to the research but not yet described in published literature, software must be made available to editors and reviewers. We strongly encourage code deposition in a community repository (e.g. GitHub). See the Nature Portfolio guidelines for submitting code & software for further information.

Data

Policy information about availability of data

All manuscripts must include a data availability statement. This statement should provide the following information, where applicable:

- Accession codes, unique identifiers, or web links for publicly available datasets
- A description of any restrictions on data availability
- For clinical datasets or third party data, please ensure that the statement adheres to our policy

The GWAS summary statistics generated in this study are available via GWAS Catalog (https://www.ebi.ac.uk/gwas/downloads/summary-statistics) under study accession identifiers GCST90044841, GCST90044842, GCST90044843, GCST90044844, GCST90044845 and GCST90044846. Summary GWAS statistics are also available at the Japanese Multi Omics Reference Panel (jMORP) website (https://jmorp.megabank.tohoku.ac.jp/gwas/; ID TGA000010).
Individual genotyping results and other cohort data used for the association study are stored in Tohoku Medical Megabank Organization. In response to reasonable requests for these data (contact us at dist@megabank.tohoku.ac.jp), we will share the stored data after assembling the data set after approval of the Ethics Committee and the Materials and Information Distribution Review Committee of Tohoku Medical Megabank Organization.

Source data underlying box plots shown in figures are provided in Supplementary Data 6.

Field-specific reporting

Please select the one below that is the best fit for your research. If you are not sure, read the appropriate sections before making your selection.

☒ Life sciences ☐ Behavioural & social sciences ☐ Ecological, evolutionary & environmental sciences

For a reference copy of the document with all sections, see nature.com/documents/nr-reporting-summary-flat.pdf

Life sciences study design

All studies must disclose on these points even when the disclosure is negative.

Sample size	We included the samples who were collected by two independent cohort studies as much as possible.
Data exclusions	We excluded samples according to the standard quality controls (QC) procedure of GWAS. The details of QCs are described in the Methods.
Replication	We performed GWAS in the two independent Japanese cohorts of the Tohoku Medical Megabank Organization. We utilized the CommCohort Study for the Discovery Stage and the BirThree Cohort Study for the Validation Stage. We basically considered P-values as the measurement of reproducibility; however, due to different sample sizes, we also evaluated the directional consistency and correlation in the effect sizes of identified variants between the Discovery Stage and the Validation Stage.
Randomization	Not applicable in our study design.
Blinding	Not applicable.

Reporting for specific materials, systems and methods

We require information from authors about some types of materials, experimental systems and methods used in many studies. Here, indicate whether each material, system or method listed is relevant to your study. If you are not sure if a list item applies to your research, read the appropriate section before selecting a response.

Materials & experimental systems

n/a	Involved in the study	
☒	☐	Antibodies
☒	☐	Eukaryotic cell lines
☒	☐	Palaeontology and archaeology
☒	☐	Animals and other organisms
☐	☒	Human research participants
☒	☐	Clinical data
☒	☐	Dual use research of concern

Methods

n/a	Involved in the study	
☒	☐	ChIP-seq
☒	☐	Flow cytometry
☒	☐	MRI-based neuroimaging

Human research participants

Policy information about studies involving human research participants

Population characteristics	This information is described in Supplementary Table 1.
Recruitment	This information is described in the Method.
Ethics oversight	This information is fully described in the Method.

Note that full information on the approval of the study protocol must also be provided in the manuscript.
